# Temporal Changes in Nuclear Envelope Permeability during Semi-Closed Mitosis in *Dictyostelium* Amoebae

**DOI:** 10.3390/cells12101380

**Published:** 2023-05-13

**Authors:** Kristina Mitic, Irene Meyer, Ralph Gräf, Marianne Grafe

**Affiliations:** Department of Cell Biology, University of Potsdam, Karl-Liebknecht-Str. 24-25, 14476 Potsdam, Germany

**Keywords:** *Dictyostelium*, nuclear envelope, nuclear pore complex, centrosome, mitosis

## Abstract

The Amoebozoan *Dictyostelium discoideum* exhibits a semi-closed mitosis in which the nuclear membranes remain intact but become permeabilized to allow tubulin and spindle assembly factors to access the nuclear interior. Previous work indicated that this is accomplished at least by partial disassembly of nuclear pore complexes (NPCs). Further contributions by the insertion process of the duplicating, formerly cytosolic, centrosome into the nuclear envelope and nuclear envelope fenestrations forming around the central spindle during karyokinesis were discussed. We studied the behavior of several *Dictyostelium* nuclear envelope, centrosomal, and nuclear pore complex (NPC) components tagged with fluorescence markers together with a nuclear permeabilization marker (NLS-TdTomato) by live-cell imaging. We could show that permeabilization of the nuclear envelope during mitosis occurs in synchrony with centrosome insertion into the nuclear envelope and partial disassembly of nuclear pore complexes. Furthermore, centrosome duplication takes place after its insertion into the nuclear envelope and after initiation of permeabilization. Restoration of nuclear envelope integrity usually occurs long after re-assembly of NPCs and cytokinesis has taken place and is accompanied by a concentration of endosomal sorting complex required for transport (ESCRT) components at both sites of nuclear envelope fenestration (centrosome and central spindle).

## 1. Introduction

During mitosis, eukaryotic cells establish a bipolar spindle with two spindle poles and microtubules connecting each pole to the kinetochores at the two sister chromatids. In all organisms possessing centrosomes as the main microtubule organizing centers, the spindle poles are built by these centrosomes after their duplication.

As centrosomes are usually cytosolic organelles and chromosomes are inside the nucleus, tubulin, spindle assembly factors, and mitotic regulators need to obtain access to the nuclear interior in order to allow spindle formation. In higher eukaryotes this is achieved by complete nuclear envelope (NE) breakdown during prophase (open mitosis) [1]. In contrast, many unicellular eukaryotes, such as budding yeast, retain an intact NE during the entire cell cycle (closed mitosis) [1]. Here, the centrosomes (often called spindle pole bodies) are permanently inserted in the nuclear envelope and all proteins required for spindle assembly and chromosome segregation enter the nuclear interior via directional nuclear transport through the nuclear pore complexes (NPCs). Between these two extremes, there are many intermediate forms of mitosis throughout all eukaryotic supergroups [2] in which the nuclear envelope remains but exhibits an unrestricted permeability for large molecules either by fenestration or partial disassembly of NPCs or both. These in-between forms of mitosis can be so different that it is misleading to subsume them altogether with the term “semi-open”. They range from rather open forms, in which the nuclear envelope no longer contains functional NPCs and is perforated by huge fenestrae, for example, in the *Drosophila* embryo [3], to fully intact mitotic nuclear envelopes, in which only a dissociation of certain NPC components is sufficient to relieve the permeability barrier for large proteins, for example, in the filamentous fungus *Aspergillus nidulans* [4]. To stress their more closed character, we prefer to call the latter forms “semi-closed” instead of “semi-open”. In a recent study we could show that *Dictyostelium* amoebae exhibit a semi-closed mitosis, whereby the nuclear envelope becomes permeabilized by a partial disassembly of NPCs in a manner highly reminiscent of *Aspergillus nidulans* [5].

Yet in *Dictyostelium* amoebae, there may exist further processes contributing to nuclear envelope permeabilization during mitosis. One is associated with centrosome duplication which involves the formation of fenestrae within the nuclear envelope harboring the mitotic centrosomes. During interphase, the centrosome is a cytosolic, nucleus-associated body (NAB) [6]. It consists of a core structure with three major layers surrounded by a corona, which nucleates microtubules during interphase. Centrosome duplication starts at the G2/M transition with the dissociation of the corona. In early prophase, the remaining core structure approaches the nuclear envelope leading to membrane deformation and subsequent integration of the core structure into a fenestra of the nuclear envelope. Although ultrastructural analyses revealed no considerable space between the nuclear envelope and the embedded mitotic centrosome [7], it cannot be excluded that the centrosomal fenestrae also serve as a portal of entry for the proteins required for spindle assembly. In early prometaphase, the central core layer disappears, and the two outer layers start to separate and organize the intra-nuclear mitotic spindle. During karyokinesis in telophase, a further opening of the nuclear envelope occurs at the constriction site between the two dividing daughter nuclei. In late telophase, the central core layer re-appears, and the centrosomes leave the nuclear envelope towards its cytosolic face. We have presented evidence that the closure of the fenestrae at the nuclear envelope at both the poles and at the abscission sites between both daughter nuclei involves the AAA-ATPase DdSpastin, the HeH-family protein Src1, and the ESCRT complex [8]. Our data from *Dictyostelium* amoebae and the data obtained in animal cells by others are in line with the requirement of the inner nuclear membrane protein Src1 for recruitment of the ESCRT complex and DdSpastin to the fenestrae [8,9,10,11,12]. While DdSpastin severs the spindle microtubules that still penetrate the fenestrae, the ESCRT complex closes the membrane gaps through its membrane-shaping activity.

Fenestrae harboring the spindle pole body (SPB) have been characterized in both budding yeast and fission yeast. In budding yeast, a new fenestra is formed upon insertion of the duplication plaque, an SPB precursor built during the duplication process [13]. In fission yeast, the duplicated SPBs enter fenestrae in the nuclear envelope in G2 prior to mitosis and are expelled into the cytoplasm again in late mitosis [14]. In both fungi, these fenestrae are tight with regard to nuclear transport, even during their formation. Yet careful studies in fission yeast revealed that there is a controlled local nuclear envelope breakdown process even during closed mitosis, whereby leakage of material from both separating daughter nuclei is prevented [15]. Interestingly, the insertion of SPBs into the nuclear envelope shares many similarities with the interphase insertion of new NPCs [16,17]. Both processes require membrane-shaping proteins and a fusion event between the inner and outer nuclear membranes, and they end up with an inserted large protein complex flanked by a highly curved membrane. A relationship between NPCs and centrosomes is also underscored by centrosome-related functions of NPC proteins, such as adequate pericentrin localization at the centrosome and normal microtubule organization (Gle1), correct centrosome segregation and centriole maturation (Nup62), centrosome association with the NE at mitotic entry (Nup133), or chromosome segregation (Nup188) [18,19,20,21,22].

In this study, we set out to elucidate the timing of events in the regulation of nuclear envelope permeability during mitosis in *D. discoideum*, particularly with respect to the behavior of the centrosome and nuclear pore complexes.

## 2. Materials and Methods

### 2.1. Cloning, Strains, and Vectors

Knock-in vectors were built according to Mitic et al. (2022) [5]. Primers are listed in the Supplementary Materials, Table S1. For the C-terminal GFP knock-in vector of CP75, the plasmid pIS1121 [23] was used, and for the C-terminal Neon knock-in constructs of the ESCRT proteins (AlxA, CHMP7, Vps4), the plasmid pIS1272 [5] was used. For the CP75-mRuby knock-in construct, GFP was replaced by a codon-optimized version of mRuby2 [24] and the blasticidin cassette was replaced by a hygromycin cassette [25]. The Cep192-mRuby knock-in was cloned the same way using the vector pIS1155 [26]. For the CP39-Cherry knock-in construct, the BioH6 cassette of the vector pIS1362 [26] was replaced by DdmCherry [27]. For the Nup62-mScarlet knock-in construct, mNeon of the Nup62-Neon knock-in vector [5] was replaced by mScarlet [25]. In order to create the GFP-TubA construct, α-tubulin with an N-terminal actin6 promoter/GFP/polylinker cassette [26] was cloned into the pLPBLP vector [28]. For the Tdtomato construct, the coding sequence [29] was codon-optimized and custom synthesized with the addition of an N-terminal NLS [30] (GeneArt Gene Synthesis from Thermo Fisher Scientific, Waltham, MA, USA). The construct was cloned between the KpnI and NsiI restriction sites of the C-terminal GFP expression vector p1ABsr8 [31].

All knock-in plasmids were linearized by restriction enzyme digestion prior to transformation in *Dictyostelium* cells (Supplementary Materials, Table S2) [5,8].

In order to generate strains coexpressing two different fluorescence tags (either knock-in or overexpression) conferring blasticidin resistance, the blasticidin cassette of the knock-in strains or the GFP-TubA strain was removed using the Cre-Lox system according to [32].

### 2.2. Cell Culture

HL5c medium (Formedium, Hunstanton, UK) supplemented with sterile filtered glucose was used. Clones were selected with 10 µg/mL G418 or 4 µg/mL Blasticidin S or 50 µg/mL hygromycin, respectively. Cells were grown in adherent culture using tissue culture flasks.

### 2.3. Microscopy

For immunofluorescence microscopy, Neon-fusion protein expressing cells were fixed with glutaraldehyde and labeled with antibodies as described previously [33], and labeled with monoclonal rat YL1/2 antibody directed against α-tubulin [34] and anti-rat AlexaFluor 594 and DNA was stained with Hoechst 33324 (both from Thermo Fisher Scientific, Darmstadt, Germany). Light microscopy, image processing, and deconvolution were performed on an AxioObserver System (Carl Zeiss, Jena, Germany) with Zeiss ZEN 2012 (blue edition) software as described recently [35].

Live-cell imaging was performed essentially as described earlier [36]. Imaging was performed according to [5], using a ZEISS Cell Observer SD confocal microscope (Carl Zeiss Microscopy GmbH, Jena, Germany) equipped with the Yokogawa Spinning Disk Unit CSU-X1 and 2 Evolve EM-CCD cameras (Photometrics, Tucson, AZ, USA) with a LCI Plan-Neofluar 63×/1.3 Imm Korr DIC objective and Zeiss AxioVision Rel. 4.9.1 software. Z-stack settings were set to 9–13 slices per stack and recorded every 10–15 s.

For videos of living cells showing centrosomes or nuclear envelopes, only one plane of the Z-stack is shown, i.e., the focus plane in the case of centrosomes and the equatorial plane of the nucleus, respectively. For nuclear markers, maximum intensity projections are presented. Fluorescence intensity measurements were conducted using Fiji. For quantification of the fluorescence signal, non-deconvolved immunofluorescence images were used. A freehand line was drawn on the mitotic nuclear envelope and the intensity value was measured as described [5]. Values were analyzed using standard statistic programs (Microsoft Excel/LibreOffice Calculator). Corresponding results are shown in diagrams or tables.

## 3. Results

### 3.1. Monitoring Nuclear Envelope Permeabilization versus Spindle Formation

In our earlier work, we used GFP-Cenp68 as a marker to monitor the permeability of the nuclear envelope during mitosis in living cells [37,38]. Cenp68 is a centromeric protein of unknown function that when overexpressed as a GFP fusion protein accumulates in the nucleus. Excess protein diffuses into the cytosol at the onset of mitosis, while only the centromere-bound portion remains in the kinetochore region [38]. Although the behavior of GFP-Cenp68 revealed that permeabilization of the nuclear envelope does take place during mitosis, this protein was not an ideal marker to monitor the dynamics of permeabilization and restoration of tightness after mitosis, since it could not be excluded that the timing of events was affected by an uncharacterized binding of Cenp68 to chromatin. This suspicion was supported by live observation by marsRFP-tagged NLS-CP224ΔC, a marker earlier used to show the functionality of the NLS of the *Dictyostelium* lamin NE81 [30]. This fusion protein accumulates in the nucleus during interphase and is also released from the nucleus during mitosis but its re-appearance in daughter nuclei was delayed compared with GFP-Cenp68, as shown in cells expressing marsRFP-NLS-CP224ΔC together with GFP-α-tubulin. Here, the nuclear marker did not re-appear in the nucleus directly after the completion of cytokinesis (Figure S1 and Video S1). Although the CP224ΔC fragment displayed an even distribution in the cytosol when expressed without an NLS [31], we could again not exclude that nuclear accumulation of marsRFP-NLS-CP224ΔC after mitosis was affected by uncharacterized activities of the protein. Therefore, we switched to an established nuclear permeabilization marker [39] to continue our study of this process in *Dictyostelium*. We used NLS-TdTom, an NLS-tagged version of two tandem copies of the red-fluorescent protein tomato.

**Figure 1:**
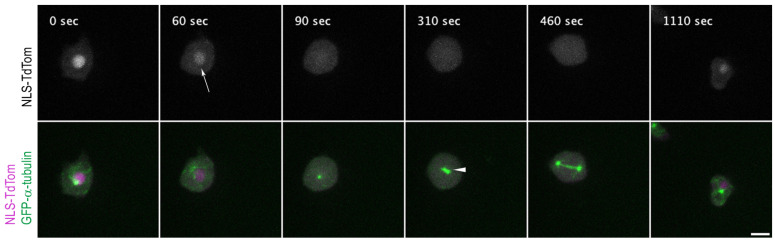
NLS-TdTom is only inside the nucleus during interphase. Selected time points of Video S2. NLS-TdTom (magenta) diffuses over the whole cell upon permeabilization of the nucleus at the onset of mitosis (60 s, arrow). Cells co-expressing GFP-α-tubulin (green) are observed to show the mitotic progression. The nuclear marker is diffused completely before the centrosome duplication (arrowhead) becomes visible (310 s). After cytokinesis, NLS-TdTom is located back to the nucleus (1110 s). Bar 5 µm.

NLS-TdTom has a size of ~60 kDa, which is sufficiently large to make sure that the marker can accumulate in the nucleus only via Ran-dependent transport through NPCs. First, we expressed NLS-TdTom again with GFP-α-tubulin. Live-cell imaging of this strain nicely revealed that nuclear envelope permeabilization starts in prophase and that the nuclear marker diffuses completely before centrosome duplication becomes visible, essentially confirming the results obtained with marsRFP-NLS-CP224ΔC (Figure 1 and Figure S1, Video S2).

### 3.2. Monitoring Nuclear Envelope Behavior versus Permeabilization and Centrosome Duplication

**Figure 2:**
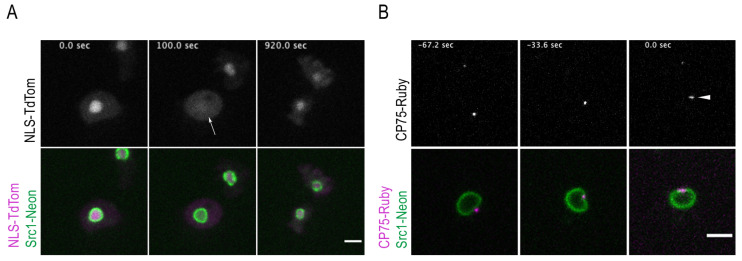
Nuclear envelope permeabilization and centrosome duplication occur at the onset of mitosis. Selected time points of Videos S3 and S4 are shown. (**A**) NE marker Src1-Neon knock-in (green) with the NE permeabilization marker NLS-TdTom (magenta). The nuclear envelope is preserved during the entire cell cycle, while NLS-TdTom diffuses into the cytosol (arrow at 100 s) and is restored long after cytokinesis (920 s). (**B**) Cell with Src1-Neon knock-in (green) and centrosomal component CP75-Ruby knock-in (magenta). The centrosome moves from a perinuclear position (−67.2 s) into the nuclear envelope (−33.6 s) before it duplicates (arrowhead at 0 s). Bars 5 µm.

We then used NLS-TdTom to transform a Src1-Neon knock-in strain (Src1-Neon-ki), which possesses a green fluorescent nuclear envelope [8]. As in all our knock-in strains in this work, expression is driven by the endogenous promoter and the endogenous copy of the respective protein (here Src1) is replaced by the tagged one. The resulting strain nicely shows that the nuclear envelope is preserved during the entire cell cycle and especially throughout mitosis while NLS-TdTom diffuses into the cytosol at the onset of mitosis and becomes re-accumulated in the nucleus only after cytokinesis (Figure 2A, Video S3).

Next, we set out to visualize centrosome insertion into the nuclear envelope in living cells. Therefore, we transformed the Src1-Neon-ki strain with a CP75-Ruby knock-in construct (CP75-Ruby-ki) to generate a cell line with green fluorescent nuclear envelopes and red fluorescent centrosomes. CP75 is a member of the central core layer and dissociates from the mitotic centrosomes right after their duplication into two entities [40]. Live-cell imaging clearly showed that centrosome duplication occurs approximately 1 min after the centrosome has moved from a perinuclear, cytosolic position into the nuclear envelope, reflecting centrosome insertion (Figure 2B, Video S4). This result was confirmed in a parallel strain, in which the Src1-Neon-ki strain was transformed with a CP39-Cherry knock-in construct. CP39 is a member of the central layer of the centrosomal core structure and is known to dissociate prior to CP75 upon centrosome duplication, i.e., it leaves the mitotic centrosome prior to splitting of the core structure into two separate entities [40]. Live-cell imaging revealed once more that centrosome insertion occurs prior to centrosome duplication, since in mitotic cells a single CP39 spot was visible in line with the nuclear envelope before CP39 started to disappear (Figure S2, Video S5). Centrosome duplication and behavior throughout all mitotic stages was visualized in a strain carrying a Cep192-Ruby knock-in and a Nup210-Neon knock-in. Cep192 is a member of the centrosomal outer core layers and, like the transmembrane Nup210, is present at the dividing nuclei throughout mitosis. The Cep192-Ruby/Nup210-Neon strain reveals that Nup210 is concentrated at the fenestrae harboring the mitotic centrosomes and it lasts there until late telophase when the centrosome is positioned towards the cytosolic side of the nuclear envelope again (Figure S3A and Video S6).

**Figure 3:**
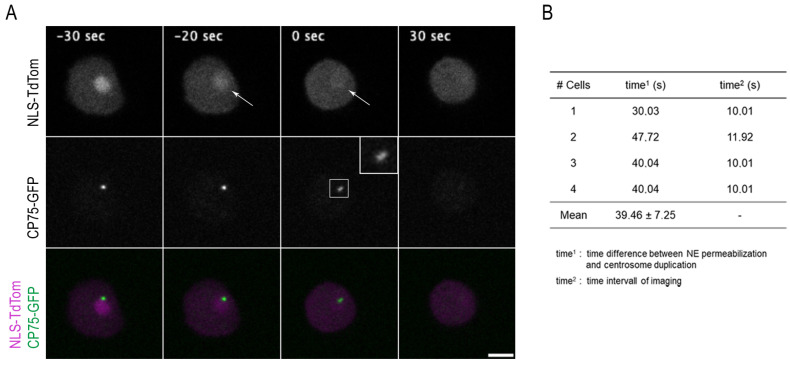
NLS-TdTom diffuses into the cytosol prior to centrosome splitting. (**A**) Selected time points from live-cell imaging of a strain expressing CP75-GFP (green) knock-in together with the NE permeabilization marker NLS-TdTom (magenta). NLS-TdTom is located within the nucleus during interphase (−30 s) and diffuses rapidly into the cytosol upon permeabilization (arrow at −20 s and 0 s) while CP75-GFP is still located at the mitotic centrosome only until the splitting of the core structures at time point 0 s (indicated by zoomed inset). Series of frames based on confocal spinning disk live-cell microscopy were selected from a movie (z-distance of 0.5 µm, recorded with a time lapse of 10 s). Only 1 slice out of a 10-layer Z-stack is shown, respectively. Bar 5 µm. See also Video S7. (**B**) Time resolution between the first NE permeabilization and the duplication of the mitotic centrosome (time^1^) and the corresponding time interval of image acquisition (time^2^). Centrosome duplication starts approximately 39 ± 7.3 s (mean and standard deviation for *n* = 4) after permeabilization of the nuclear envelope.

In a further strain, we studied nuclear permeabilization with respect to centrosome duplication. Again, we chose CP75 as the centrosomal marker and created a knock-in construct with a C-terminal GFP tag (CP75-GFP-ki) in order to allow two-color studies together with red-fluorescent NLS-TdTom. Live-cell imaging of this strain revealed that centrosome duplication starts approximately 39 ± 7.3 s after permeabilization of the nuclear envelope, i.e., start of NLS-TdTom diffusion into the cytosol (Figure 3A, Video S7), which fits nicely to the results obtained earlier with the marsRFP-NLS-CP224ΔC/GFP-α-tubulin strain (Figure S1).

### 3.3. Monitoring NPC Disassembly versus Centrosome Duplication

**Figure 4:**
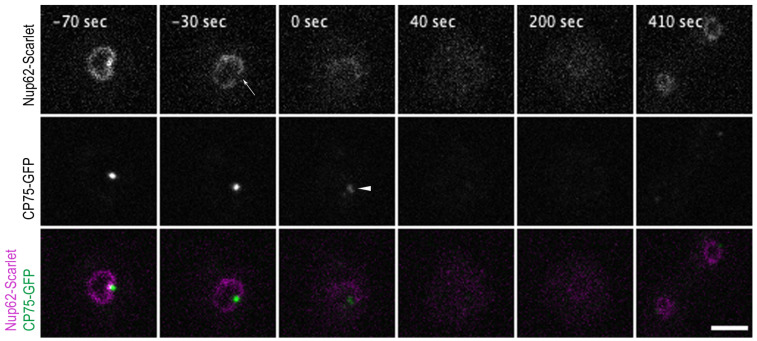
Nup62-Scarlet disassembly from NPCs precedes centrosome splitting at the onset of mitosis. Selected time points from a live-cell imaging (Video S8) show a cell co-expressing the Nup62-Scarlet (magenta) and CP75-GFP (green) knock-in construct. CP75-GFP is still located at the mitotic centrosome when Nup62-Scarlet starts to dissociate from the NPCs (−30 s, arrow). The time point of 0 s indicates the centrosome splitting which appears as 2 dots (arrowhead). Nup62 dissociation occurred approx. 33 ± 8.4 s (mean and standard deviation for *n* = 9) prior to centrosome duplication (see Table S3). CP75-GFP leaves the centrosome after the duplication process (40 s). Image stacks of 10 layers (z-distance of 0.5 µm) were recorded with a 10 s time lapse and only 1 focal plane is presented. Bar 5 µm.

As our recent study on the mitotic behavior of nuclear pore complex components revealed a partial mitotic disassembly of the NPC suggesting that this contributes to nuclear envelope permeabilization [5], we studied the timing of centrosome duplication monitored by CP75-GFP with respect to partial NPC disassembly monitored by Nup62-Scarlet localization. Thus, the CP75-GFP knock-in strain was transformed with a Nup62-Scarlet knock-in construct and living mitotic cells were imaged. Our videos showed that Nup62 labeling of the nuclear envelope started to decrease approximately at the same time when the centrosome signal moved from a lateral position outside the nuclear envelope to a position where it integrated into the nuclear envelope. This occurred approximately 33 ± 8.4 s prior to centrosome duplication (Figure 4, Table S3, Video S8), which corresponds well to the timing of NLS-TdTom diffusion into the cytosol described above.

### 3.4. Monitoring NPC Disassembly versus Nuclear Envelope Permeabilization

**Figure 5:**
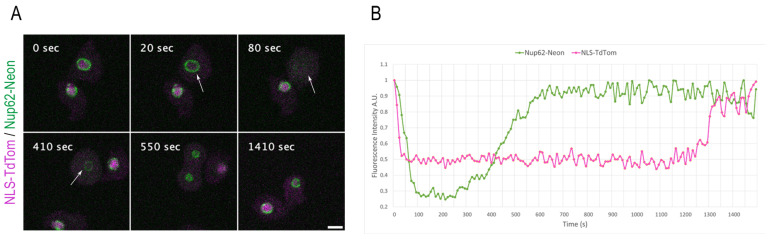
NE permeabilization versus Nup62 disassembly during mitosis. (**A**) Selected time points from live-cell imaging (Video S10) of a strain co-expressing the Nup62-Neon knock-in construct (green) and the NE permeabilization marker NLS-TdTom (magenta). NLS-TdTom leaves the nucleus at the onset of mitosis while Nup62-Neon is still present at the NE (arrow at 20 s). Nup62-Neon completely disassembles from the NE (arrow at 80 s) before it reappears in metaphase (arrow at 410 s). Out of a 12-layer image stack (z-distance 0.5 µm), only the focus plane is presented, respectively. Images were recorded with a 10 s time lapse. Bar 5 µm. (**B**) The graph presents the fluorescence intensity values for Nup62-Neon knock-in and NLS-TdTom over the complete cell division in (**A**).

To study whether loss of NLS-TdTom was indeed synchronized with the partial disassembly of NPCs, we created corresponding knock-in strains expressing Nup62, Nup93, and Nup210 C-terminally tagged with green fluorescent Neon. While Nup210 remains at the NE during the entire mitosis as shown above (Figure S3 and Videos S6 and S9), the FG-repeat protein Nup62 and the inner ring protein Nup93 dissociate from the NE during prophase [5]. These strains were transformed with NLS-TdTom and their mitotic behavior was imaged by spinning disk microscopy. Among the dissociating Nups, we focused on Nup62 since among all Nups tested this one exhibited the longest period of absence at mitotic nuclei and, thus, appears as the most suitable marker to monitor NPC restoration in late mitosis. Live-cell imaging indicated a gradual loss of Nup62 during prophase, indicating that disassembly occurred not in complete synchrony at all individual NPCs. Yet a side-by-side comparison of parallel intensity measurements of Nup62-Neon fluorescence at the nuclear envelope and the nuclear interior (NLS-TdTom) showed that permeabilization of the nuclear envelope was synchronized with partial disassembly of the NPCs (Figure 5, Video S10). This was also shown for the inner ring NPC protein Nup93 (Figure S4, Video S11). As expected, restoration of nuclear envelope integrity, i.e., the re-concentration of NLS-TdTom in the nuclear interior of daughter cells was observed only after restoration of Nup62 fluorescence at the nuclear envelope in telophase. Interestingly, Nup93-Neon started to disappear from the nuclear envelope slightly after Nup62 and re-appeared at mitotic NPC already in late metaphase/early anaphase prior to Nup62 (Figure S5, Video S12). However, the time interval between restoration of Nup62-Neon fluorescence and NLS-TdTom fluorescence in the nuclear interior differed considerably from cell to cell, indicating that restoration of nuclear envelope integrity after karyokinesis in telophase required not only the restoration of NPC integrity.

### 3.5. Monitoring ESCRT Behavior at Nuclear Envelope Fenestrae in Late Mitosis

According to our recent studies, we expected fenestrae at the abscission sites between the two dividing nuclei [8]. In late telophase, the nuclear envelopes of the dividing nuclei are still penetrated by the central spindle. We provided evidence that spastin needs to sever the microtubules of the central spindle in order to allow closure of the fenestrae and that the latter involves the ESCRT complex [8]. In order to study the restoration of nuclear envelope integrity more precisely, we tried several ESCRT complex components as markers for fenestral closure sites and created corresponding knock-in strains expressing these components with a C-terminal Neon tag. Among the tested ones (Chmp7, Vps4, and AlxA = ALIX), AlxA yielded the strongest fluorescence and the corresponding knock-in strain was the only one suitable for live-cell imaging.

**Figure 6:**
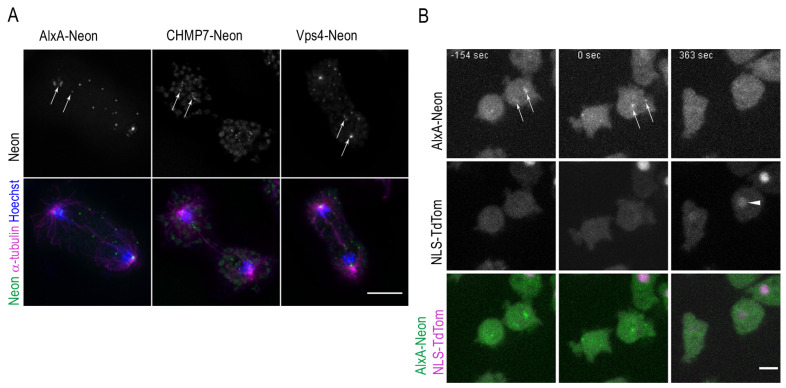
ESCRT proteins concentrate at the nuclear envelope fenestration sites in mitosis and long after cytokinesis has taken place. (**A**) Cells were fixed with glutaraldehyde and stained with Hoechst (blue) and anti-α-tubulin (magenta). ESCRT-Neon fusion proteins (green) localize at the nuclear envelope fenestration sites in telophase (arrows). A maximum intensity projection of several slices of the deconvolved Z-stack is presented. (**B**) Selected time points of Video S13 are shown with cells in cytokinesis (−154 s), after cell division (0 s), and once AlxA-Neon has disappeared together with reappearance of NLS-TdTom (arrowhead) at 363 s. Bars 5 µm.

Yet in all cases, Neon fluorescence was sufficiently strong to allow a study of their distribution in fixed late mitotic cells. In all cases, ESCRT protein spots were in agreement with localization at nuclear envelope fenestrae, both at mitotic spindle poles/centrosomes and at the abscission sites (Figure 6A, arrows). Live-cell imaging of AlxA-Neon-ki cells transformed with NLS-TdTom revealed that AlxA-Neon foci are maintained until long after completion of cytokinesis and in parallel, recovery of NLS-TdTom concentration in the nucleus also occurs only after completion of cytokinesis (Figure 6B, Video S13).

## 4. Discussion

**Figure 7:**
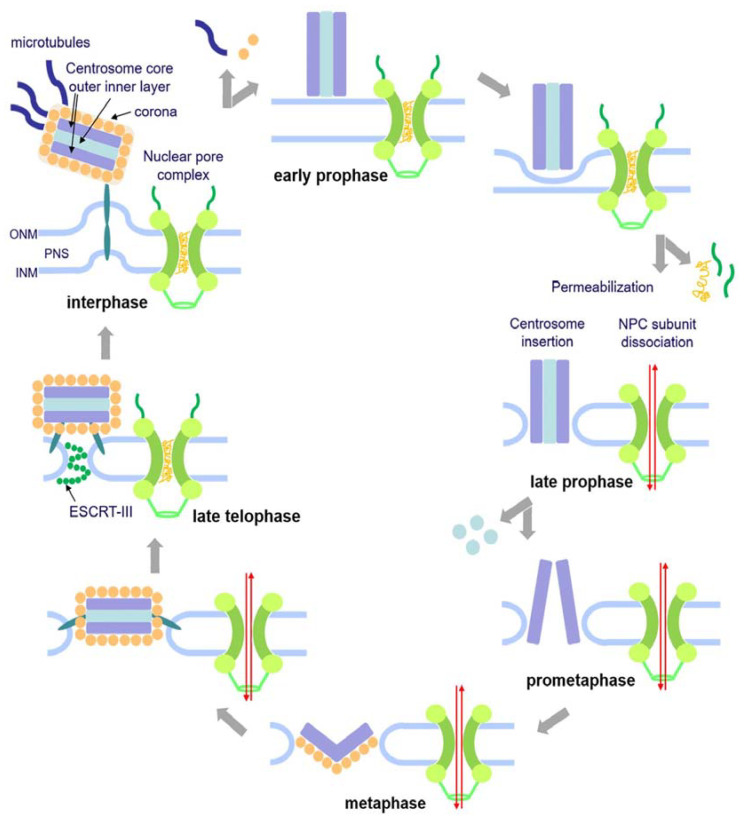
Hypothesis of centrosome insertion and nuclear envelope permeabilization in *Dictyostelium discoideum*. The onset of mitosis is characterized by the loss of the corona together with the attached microtubules in early prophase. The fusion of the inner and outer nuclear membranes forms a fenestra and allows the insertion of the mitotic centrosome into the nuclear envelope. Simultaneously, the partial disassembly of the nuclear pore complexes contributes to the permeabilization of the nuclear envelope in late prophase. Next, in prometaphase, the central layer of the core structure disappears, the mitotic centrosome splits, and the outer entities move apart. During telophase, the central core layer re-appears and the nuclear pore complexes are reassembled. The duplicated centrosomes exit their fenestrae in the nuclear envelope and the ESCRT-III complex starts re-sealing the nuclear envelope to close the fenestrae.

In this work, we show for the first time in living cells that permeabilization of the nuclear envelope in mitotic *Dictyostelium* amoebae occurs in synchrony with centrosome insertion into the nuclear envelope and partial disassembly of nuclear pore complexes. Furthermore, we showed in live cells that centrosome duplication takes place *after* its insertion into the nuclear envelope and *after* initiation of permeabilization (Figure 2). Restoration of the nuclear envelope integrity usually occurs long *after* re-assembly of NPCs has taken place and is accompanied by a concentration of ESCRT components at sites of nuclear envelope fenestration (Figure 6 and Figure 7). This process is completed only *after* completion of cytokinesis, i.e., in S-phase of the next cell cycle (a G1 phase does not appear to exist in *Dictyostelium* [41]). We interpret this delay in the following way: Directed nuclear transport recovers as soon as NPCs are restored since this transport depends only on cytosolic karyopherins, intact NPCs, and the differential localization of Ran-GAP to the cytosolic filaments of NPCs and Ran-GEF to chromatin. However, as long as fenestration of the nuclear envelope around the central spindle is still present, NLS-TdTom cannot be efficiently concentrated in the nuclear interior. Only when the fenestrae are completely closed again by the activity of the ESCRT complex does NLS-TdTom become trapped in the nucleus again. Since the closure of fenestrae via the ESCRT complex is a gradual process, NLS-TdTom re-accumulates only slowly. Observations in living cells expressing GFP-tubulin have shown in several studies that the timing of central spindle disruption in telophase varies widely relative to the progress of cytokinesis, i.e., sometimes it occurs already at the beginning of cleavage furrow formation and sometimes only shortly prior to abscission of the bridge between the dividing daughter cells (see, e.g., Video S1 in [40] or respective figures in [42,43]). Since closure of the fenestrae at the abscission site between the two daughter nuclei is physically impossible until the central spindle is severed, the widely varying time difference of reappearance of NLS-TdTom in the nucleus relative to restoration of NPCs was expected.

While it is obvious that fenestration of the nuclear envelope at the abscission site between the two daughter nuclei contributes to permeability of the nuclear envelope in addition to partial disassembly of NPCs, the situation is less clear at the fenestrae harboring the mitotic centrosomes. We still cannot judge whether integration of the centrosome into a fenestra of the nuclear envelope prior to its duplication also contributes to nuclear envelope permeabilization. This is because centrosome insertion into the nuclear envelope and loss of Nup62 (and Nup93) from the nuclear envelope occur at the same time as diffusion of the NLS-TdTom nuclear marker into the cytosol, and currently there are no specific means to block either of these processes for closer analyses. In analogy to fission yeast, where the spindle pole body (i.e., the centrosome) enters a fenestra in the nuclear envelope at the G2/M transition in a similar manner as in *Dictyostelium*, and where this process is not accompanied by permeabilization of the nuclear envelope during the insertion process, it appears likely that this situation is phenocopied in *Dictyostelium*. Yet by contrast to fission yeast, where mitosis is closed [15,44], the nuclear envelope becomes permeabilized in early mitosis by partial disassembly of NPCs as in *Aspergillus*, where the spindle pole body is permanently integrated in the nuclear envelope [4,45]. We suggest that the process of centrosome insertion into the nuclear envelope and its permeabilization is conserved in evolution of Amoebozoans such as *Dictyostelium* and fungi, i.e., centrosome insertion follows the process described for fission yeast [46,47] and nuclear envelope permeabilization occurs in a similar manner as described for *Aspergillus* [4].

*Dictyostelium* amoebae express a couple of candidate proteins that could play a role in mitotic centrosome insertion. First of all, the single *Dictyostelium* orthologue of fungal Brr6/Brl1 nuclear envelope membrane protein (Dictybase DDB_G0274715) could play a role in this process. Brr6 orthologues are expressed only in organisms undergoing a closed mitosis, such as fission yeast or budding yeast [47,48]. In both yeasts, Brr6 promotes spindle pole body insertion into the nuclear envelope [46,49]. The fact that the *Dictyostelium* genome contains a clear homologue of this protein family argues in favor of a conserved mechanism of centrosome insertion. A further interesting candidate is the NPC component Nup53, which in addition to its role at the NPC is a part of the centrosomal core structure and possesses membrane-shaping properties in animal cells [5]. In its close proximity is CP75. Our previous work in a CP75-RNAi strain suggested that the protein could play a role in insertion of the centrosome into the nuclear envelope since mitotic cells sometimes exhibited extranuclear spindles and other mitotic defects [40]. Yet further analyses in cells carrying a CP75-GFP knock-in to monitor expression levels of CP75 and the NLS-TdTom construct did exhibit failures in mitosis upon CP75-RNAi treatment; however, there were no indications of defects in centrosome insertion or nuclear envelope permeabilization (K. Mitic, unpublished data). In analogy to fission yeast, we also expect a role of Sun1 in the centrosome insertion process. Similar to its fission yeast orthologue Sad1 [50], Sun1 is concentrated at the spindle poles and forms a ring-like structure around the embedded mitotic centrosomes [51]. A similar localization pattern is observed for a couple of NPC components that persist at the nuclear envelope during mitosis such as Nup210 or TPR [5]. This is in line with the molecular and mechanistic similarities between NPC insertion and spindle pole insertion in yeasts [47,48].

Future experiments will be directed towards a further elucidation of the interactions between NPC components, nuclear envelope transmembrane proteins, and centrosome components during the centrosome insertion process and also the reciprocal centrosome extrusion process at the end of mitosis.

## Data Availability

Genomic data on the genes are available at https://dictybase.org accessed on 31 March 2023. Further original data are available upon request to the corresponding author.

## Supplementary Materials

The following supporting information can be downloaded at: https://www.mdpi.com/article/10.3390/cells12101380/s1, **Table S1**: List of primer sequences used for knock-in vector construction; **Table S2**: *Dictyostelium discoideum* strains used in this study; **Table S3**: Time resolution of Nup62 dissociation with respect to CP75 duplication; **Figure S1**: mRFP-NLS-CP224∆C leaves the nucleus at the onset of mitosis; **Figure S2**: CP39-Cherry inserts into the nuclear envelope at the onset of mitosis and dissociates before core splitting; **Figure S3**: NE permeabilization versus Nup210 localization during mitosis; **Figure S4**: NE permeabilization versus Nup93 dissociation during mitosis; **Figure S5**: Dynamic changes of the NPCs during mitosis; **Video S1**: mRFP-NLS-CP224∆C leaves the nucleus at the onset of mitosis; **Video S2**: NLS-TdTom leaves the nucleus at the onset of mitosis; **Video S3**: The nuclear envelope is preserved during the entire cell cycle and gets permeable at the onset of mitosis; **Video S4**: The centrosome inserts into the nuclear envelope at the onset of mitosis; **Video S5**: CP39-Cherry inserts into the nuclear envelope at the onset of mitosis and dissociates before core splitting; **Video S6**: Nup210 and Cep192 localization during mitosis; **Video S7**: NLS-TdTom diffuses into the cytosol prior to centrosome splitting; **Video S8**: Nup62-Scarlet disassembly from NPCs precedes centrosome splitting at the onset of mitosis; **Video S9**: NE permeabilization versus Nup210 localization during mitosis; **Video S10**: NE permeabilization versus Nup62 dissociation during mitosis; **Video S11**: NE permeabilization versus Nup93 dissociation during mitosis; **Video S12**: Dynamic changes of the NPCs during mitosis; **Video S13**: ESCRT proteins concentrate at the nuclear envelope fenestration sites in mitosis and long after cytokinesis has taken place.
