# Supplementary material for: Temporal Changes in Nuclear Envelope Permeability during Semi-Closed Mitosis in Dictyostelium Amoebae
Source: Cells. 2023 May 13;12(10):1380. doi: 10.3390/cells12101380 (PMC10216430; doi:10.3390/cells12101380)
Supplement: Supplementary file 1 [file cells-12-01380-s001.zip › Supplementary Materials Resub.pdf]

Supplementary Materials:
The following supporting information can be downloaded at: www.mdpi.com/xxx/s1,

Table S1: **List of primer sequences used for knock-in vector construction**

Gene	direction	Primer sequence (5′-3′)
CP75	f	AAAGGTACCCAAATAATAAATTCATTTCAAAAAGACC
	r	GGGGAATTCAAATATATCATTTCTTGTTTTAAAAGGAG
	f	GGGGCTGCAGCTTTTAAAACAAGAAATGATATATTTTAAATAAC
	r	GCATGGATCCAGGCTTTATTTCAAGTTTGG
AlxA	f	GATCGGTACCCACAAATCAAAAATCAAAACAAGG
	r	GATCGAATTCATAATGTTTATTATTTGAATTATAAGG
	f	GATCCTGCAGCAGGTTCATTTGGTGAAACGG
	r	GATCGGATCCCGTCAACAGTGTTATTAAAGAC
CHMP7	f	GATCGGTACCACTTTGAAACAACAAGAATCG
	r	GATCGAATTCAATTAATTCACTTGTTTTTTG
	f	GATCCTGCAGCCATTTTCTATCAAAGATTGCACC
	r	GATCGGATCCGATTGGTAAATTAGCATGGCC
Vps4	f	GAAAGGTACCTGAAACAATTGTTTGAGATGG
	r	AATTGTCGACTACACCATCTTGCCCAAAATC
	f	TTTTCCCTGCAGGTAACATCATTATCAAAAG
	r	GATCGGATCCCAAATTGAAAATGAAAAATTG

F, forward primer; R, reverse primer

Table S2: ***Dictyostelium discoideum* strains used in this study**

NAME	DESCRIPTION	
**AX2**	Wildtype	
**IS527**	Nup93-Neon knock-in	Mitic et al. 2022
**IS535**	Nup210-Neon knock-in	Mitic et al. 2022
**IS675**	Nup62-Neon knock-in	Mitic et al. 2022
**IS718**	Src1-Neon knock-in	Schweigel et.al 2022
**IS931**	Cep192-Ruby knock-in/Nup210-Neon knock-in	this study
**IS922**	Src1-Neon knock-in /CP75-Ruby knock-in	this study
**IS916**	AlxA-Neon knock-in /NLS-TdTomato	this study
**IS883**	Nup93-Neon knock-in /Nup62-Scarlet knock-in	this study
**IS838**	CP75-GFP knock-in/NLS-TdTomato	this study
**IS836**	CP75-GFP knock-in/Nup62-Scarlet knock-in	this study
**IS799**	Vps4-Neon knock-in	this study
**IS773**	CP39-Cherry knock-in/Src1-Neon knock-in	this study
**IS759**	NLS-TdTomato/Src1-Neon knock-in	this study
**IS742**	NLS-TdTomato/ Nup210-Neon knock-in	this study
**IS731**	Nup93-Neon knock-in / NLS-TdTomato	this study
**IS730**	AlxA-Neon knock-in	this study
**IS723**	NLS-TdTomato/Nup62-Neon knock-in	this study
**IS720**	NLS-TdTomato/ GFP-α-tubulin	this study
**IS634**	CHMP7-Neon knock-in	this study
**IS626**	mRFP-NLS-CP224ΔC/ GFP-α-tubulin	this study

Table S3: **Time resolution of Nup62 dissociation with respect to CP75 duplication**

# Cells	time[1] (s)	time[2] (s)
1	35.82	11.94
2	47.76	11.94
3	35.82	11.94
4	23.88	11.94
5	30.03	10.01
6	30.03	10.01
7	30.03	10.01
8	40.04	10.01
9	20.02	10.01
Mean	32.60 ± 8.37	-

time[1] : time difference between Nup62 dissociation and centrosome duplication
time[2] : time interval of imaging

Figures

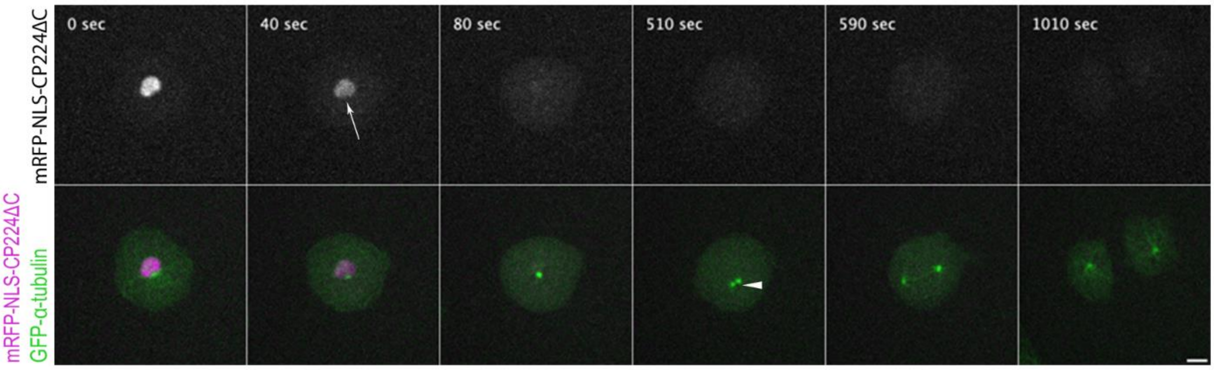

Figure S1: mRFP-NLS-CP224ΔC leaves the nucleus at the onset of mitosis.
Cells expressing the NE permeabilization marker marsRFP-NLS-CP224ΔC (magenta) and GFP-α-tubulin (green) to show mitotic progression. marsRFP-NLS-CP224ΔC diffuses into the cytosol upon permeabilization of the nucleus (40 sec, arrow). The nuclear marker is diffused completely before centrosome duplication (arrowhead) becomes visible (510 sec). Selected time points of a maximum intensity projection of Video S1 (7-layered z-stack with z-distance 0.21 µm) are shown. Bar 5 µm.

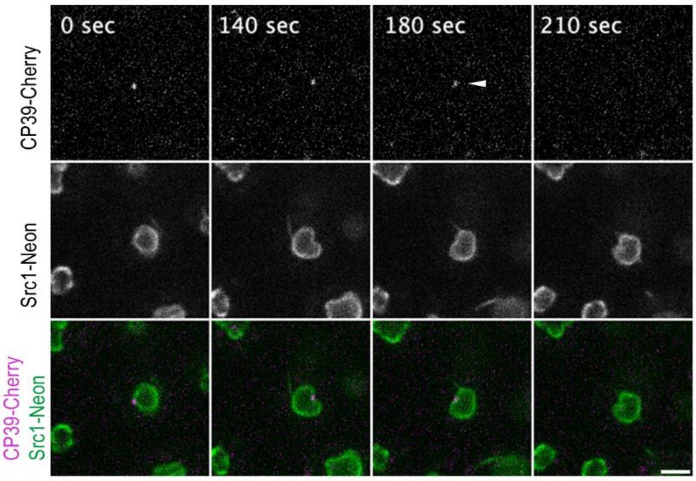

Figure S2: CP39-Cherry inserts into the nuclear envelope at the onset of mitosis and dissociates before core splitting. Selected time points of Video S5. Cells expressing CP39-Cherry knock-in (magenta) with Src1-Neon knock-in (green). CP39-Cherry locates at the cytosolic site of the NE during interphase (0 sec) before it moves into the NE (140 sec) and dissociates (arrowhead at 180 sec) completely from the mitotic centrosome (210 sec) before centrosome splitting. Time lapse imaging was performed with a 10 s time interval and z-stacks of 5 images (z-distance of 0.5 μm). Only the focus plane is presented, respectively. Bar 5 μm.

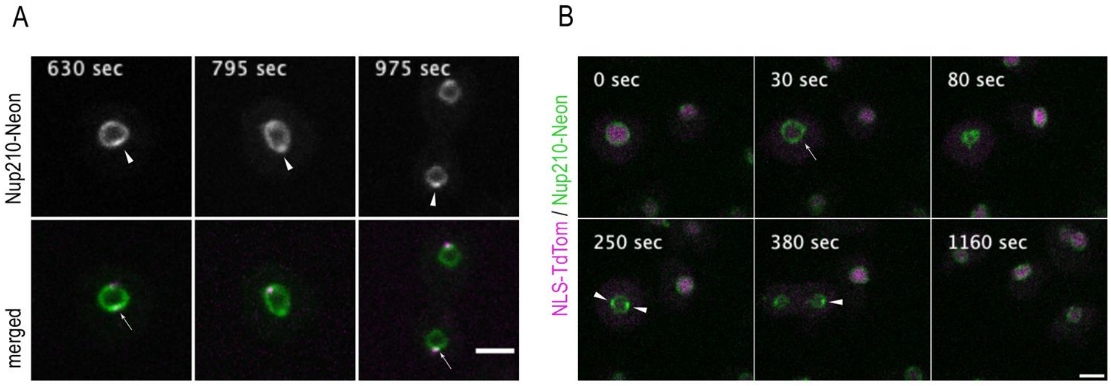

Figure S3: NE permeabilization versus Nup210 localization during mitosis. Nup210-Neon remains at the nuclear envelope throughout mitosis and is concentrated at the poles (arrowheads). Live cell microscopy of Nup210-Neon knock-in (green) cells with (A, Video S6) the centrosomal protein Cep192-Ruby knock-in (magenta) or (B, Video S9) the NE permeabilization marker NLS-TdTom (magenta). Selected time points of Videos S6 and S9 are shown. A: Cep192 is a component of the outer core layers and remains at the centrosome throughout mitosis. Co-localization with Nup210-Neon at the spindle pole sites (arrows). B: NLS-TdTom diffuses into the cytosol at the onset of mitosis (arrow at 30 sec). Bars 5 μm.

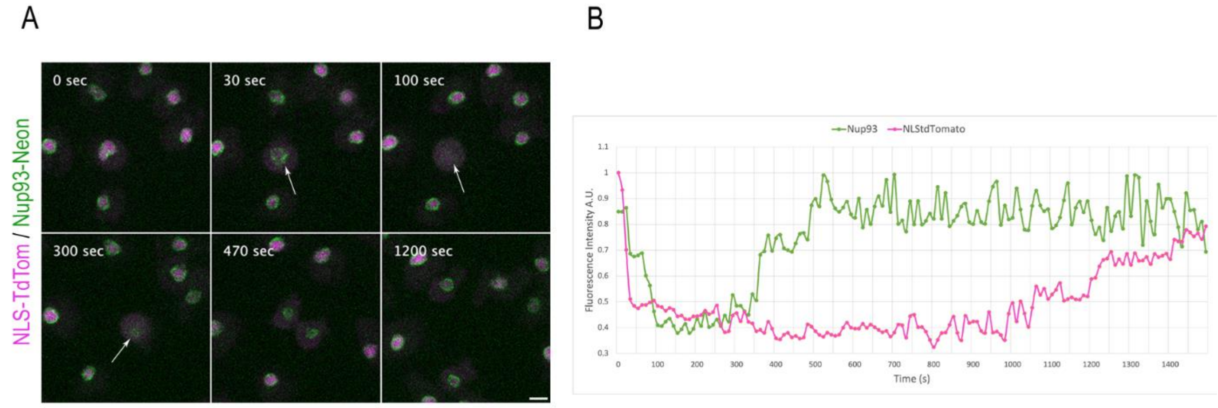

Figure S4: NE permeabilization versus Nup93 dissociation during mitosis. Live cell microscopy of Nup93-Neon knock-in (green) cells with the NE permeabilization marker NLS-TdTom (magenta). A: Selected time points of Video S11 are shown. NLS-TdTom leaves the nucleus (30 sec) at the onset of mitosis while the inner ring Nup93 is still present at the NE (arrow at 30 sec). Nup93-Neon completely dissociates from mitotic NPCs (arrow at 100 sec) before it reappears in metaphase (arrow at 300 sec) before karyokinesis. Bar 5 µm. B: The graph presents the fluorescence intensity values for Nup93-Neon knock-in and NLS-TdTom over the complete cell division. Nup93-Neon and NLS-TdTom signals decrease at the onset of mitosis. Nup93 increases before karyokinesis (350 s) whereas the NLS-TdTom signal increases after cytokinesis (1200 s).

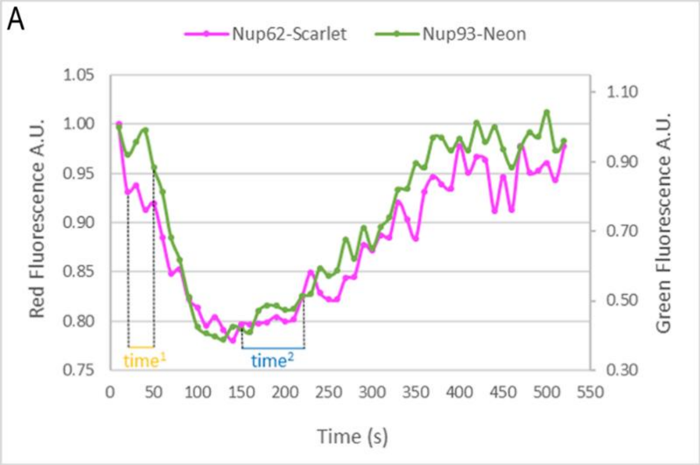

B

# Cells	time¹ (s)	time² (s)
1	12.8	51.8
2	30.0	50.0
3	30.0	40.0
4	10.0	50.0
Mean	20.7	47.8

time¹ : time difference between Nup62 and Nup93 disappearance
time² : time difference between Nup93 and Nup62 reappearance

Figure S5: Dynamic changes of the NPCs during mitosis. A: Intensity profile of the fluorescence signals of a Nup62-Scarlet knock-in and Nup93-Neon knock-in cell entering mitosis. B: Table with time difference between Nup62 disappearance compared to Nup93 disappearance (time¹) and Nup93 reappearance according to Nup62 reappearance (time²) in mitosis. The central FG-repeat protein Nup62 disappears slightly before the inner ring Nup93 from mitotic NPCs at the onset of mitosis. Nup93 reappears in late metaphase prior to Nup62 and before karyokinesis. Videos are either 9-layer image stacks (z-distance of 0.2 μm) recorded with a time lapse of 10 s or 13-layer image stacks (z-distance of 0.2 μm) recorded with a time lapse of 12.8 s.

Videos

Video S1 mRFP-NLS-CP224ΔC leaves the nucleus at the onset of mitosis. Live cell microscopy of cells expressing the NE permeabilization marker marsRFP-NLS-CP224ΔC (magenta) and GFP-α-tubulin (green) to show mitotic progression. marsRFP-NLS-CP224ΔC diffuses into the cytosol upon permeabilization of the nucleus (40 sec). The nuclear marker is diffused completely before centrosome duplication becomes visible (510 sec). Maximum intensity projection of a 7-layered z-stack with z-distance 0.21 μm. Bar 5μm.

Video S2 NLS-TdTom leaves the nucleus at the onset of mitosis. Live cell microscopy of cells expressing the NE permeabilization marker NLS-TdTom (magenta) and GFP-α-tubulin (green) to show mitotic progression. NLS-TdTom diffuses over the whole cell upon permeabilization of the nucleus at the onset of mitosis (60 sec). The nuclear marker is diffused completely before centrosome duplication becomes visible (210 sec). After cytokinesis NLS-TdTom is located back to the nucleus (1110 sec). Maximum intensity projection of a 7-layered z-stack (z-distance 0.5 μm). Bar 5μm.

Video S3 The nuclear envelope is preserved during the entire cell cycle and gets permeable at the onset of mitosis. Live cell microscopy of cells expressing Src1-Neon knock-in (green) with the NE permeabilization marker NLS-TdTom (magenta). NLS-TdTom diffuses into the cytosol (100 sec) and is restored long after cytokinesis (920 sec). Bar 5 μm.

Video S4 The centrosome inserts into the nuclear envelope at the onset of mitosis. Live cell microscopy of cells expressing Src1-Neon knock-in (green) and centrosomal component CP75-Ruby knock-in (magenta). The centrosome moves from a perinuclear position (-67.2 sec) into the nuclear envelope (-33.6 sec) before it duplicates (0 sec). Bar 5 μm.

Video S5 CP39-Cherry inserts into the nuclear envelope at the onset of mitosis and dissociates before core splitting. Live cell microscopy of cells expressing CP39-Cherry knock-in (magenta) with Src1-Neon knock-in (green). CP39-Cherry locates at the cytosolic site of the NE during interphase (0 sec) before it moves into the NE (140 sec) and dissociates (180 sec) completely from the mitotic centrosome (210 sec) before centrosome splitting. Time lapse imaging was performed with a 10 s time interval and z-stacks of 5 images (z-distance of 0.5 μm). Only the focus plane is presented, respectively. Bar 5 μm.

Video S6 Nup210 and Cep192 localization during mitosis. Live cell microscopy of cells expressing Nup210-Neon knock-in (green) with Cep192-Ruby knock-in (magenta). Nup210 is concentrated at the fenestrae harboring the mitotic centrosomes (arrows at 795 sec) and lasts until late telophase. Cep192 is a component of the outer core layers and remains at the centrosome throughout mitosis. Bar 5 μm.

Video S7 NLS-TdTom diffuses into the cytosol prior to centrosome splitting. Live cell microscopy of cells expressing CP75-GFP knock-in (green) with the NE permeabilization marker NLS-TdTom (magenta). NLS-TdTom is located within the nucleus during interphase (-48 sec) and diffuses rapidly into the cytosol upon permeabilization (-36 sec and -24 sec) while CP75-GFP is still located at the mitotic centrosome until the splitting of the core structures at time point 0 sec. Bar 5 μm.

Video S8 Nup62-Scarlet disassembly from NPCs precedes centrosome splitting at the onset of mitosis. Live cell microscopy of cells co-expressing Nup62-Scarlet knock-in (magenta) and CP75-GFP knock-in (green). CP75-GFP is still located at the mitotic centrosome when Nup62-Scarlet starts to dissociate from the NPCs (-30 sec). Time point 0 sec indicates the centrosome splitting which appears as two dots. CP75-GFP leaves the centrosome after the duplication process (40 sec). Bar 5 μm.

Video S9 NE permeabilization versus Nup210 localization during mitosis. Live cell microscopy of cells expressing Nup210-Neon knock-in (green) with the NE permeabilization marker NLS-TdTom (magenta). NLS-TdTom leaves the nucleus at the onset of mitosis while Nup210-Neon remains at the nuclear envelope throughout mitosis and is concentrated at the poles. Bar 5 μm.

Video S10 NE permeabilization versus Nup62 dissociation during mitosis. Live cell microscopy of cells expressing Nup62-Neon knock-in (green) with the NE permeabilization marker NLS-TdTom (magenta). NLS-TdTom leaves the nucleus (20 sec) at the onset of mitosis while Nup62-Neon is still present at the NE. Nup62-Neon completely disassembles from the NE (80 sec) before it reappears in metaphase (410 sec). Bar 5 μm.

Video S11 NE permeabilization versus Nup93 dissociation during mitosis. Live cell microscopy of cells expressing Nup93-Neon knock-in (green) with the NE permeabilization marker NLS-TdTom (magenta). NLS-TdTom leaves the nucleus at the onset of mitosis while the inner ring Nup93 is still present at the NE (arrows at 30 sec). Nup93-Neon completely dissociates from mitotic NPCs (arrow at 100 sec) before it reappears in metaphase (arrow at 300 sec) before karyokinesis. Bar 5 μm.

Video S12 Dynamic changes of the NPCs during mitosis. Live cell microscopy of cells expressing Nup93-Neon knock-in (green) with the Nup62-Scarlet knock-in (magenta). The central FG-repeat protein Nup62 disappears slightly before the inner ring Nup93 from mitotic NPCs at the onset of mitosis. Nup93 reappears in late metaphase prior to Nup62 and before karyokinesis. Bar 5 μm.

Video S13 ESCRT proteins concentrate at the nuclear envelope fenestration sites in mitosis and long after cytokinesis has taken place. Live cell microscopy of cells expressing AlxA-Neon knock-in (green) with the NE marker NLS-TdTom (magenta). Bar 5 μm.
